# Efficacy and safety of intradermal botulinum toxin A for post-acne erythema: a split-face randomized controlled trial

**DOI:** 10.3389/fmed.2025.1610125

**Published:** 2025-11-17

**Authors:** Li Yang, Yifei Gao, Huajuan Wu, Yunfei Li, Chenwen Li, Xueli Li

**Affiliations:** Dermatology Department, Henan Provincial People's Hospital, Zhengzhou, China

**Keywords:** post-acne erythema, botulinum toxin type A, intradermal injection, randomized controlled trial, Clinician Erythema Assessment

## Abstract

**Introduction:**

Post-acne erythema (PAE) is a common aesthetic sequela in patients with acne. Although numerous treatments for PAE have been investigated, the efficacy of intradermal botulinum toxin A (BTX-A) remains controversial. This study aimed to evaluate and compare the efficacy and safety of intradermal BTX-A versus broadband light (BBL) for treating PAE.

**Materials and methods:**

This study included 30 patients diagnosed with PAE at the Dermatology Outpatient Department of Henan Provincial People’s Hospital in China between January 2023 and July 2023. In a split-face design, one cheek of each patient was randomly assigned to receive a single intradermal injection of BTX-A (experimental group), while the other cheek received three BBL treatments at one-month intervals (control group). The Clinician Erythema Assessment (CEA) score, VISIA results, L*a*b* values, skin physiological parameters, and adverse events were compared between the two groups.

**Results:**

The two groups showed no significant differences in baseline characteristics. At the one-month follow-up, the experimental group demonstrated significantly greater reduction in CEA, erythema index (EI), a* value, and sebum secretion compared to the control group (*p* < 0.05). The reduction in sebum secretion remained significantly greater in the experimental group at both the two- and three-month follow-ups (*p* < 0.05). Additionally, the experimental group showed a significantly greater reduction in the CEA score at 2 months and in the a* value at 3 months (*p* < 0.05). Compared to baseline, both groups exhibited a declining trend in EI, transepidermal water loss (TEWL), CEA score, sebum secretion, and a* value, along with an increase in skin hydration. Pain or discomfort, assessed by the visual Analog scale, was significantly higher in the experimental group (*p* < 0.001); however, there was no significant difference in satisfaction scores between the groups.

**Conclusion:**

In this split-face study, a single session of intradermal BTX-A was superior to a multi-session BBL regimen in reducing PAE over three months, with a comparable safety profile. These findings suggest that intradermal BTX-A is a promising and viable therapeutic option for PAE, warranting further investigation in larger, long-term studies.

**Clinical trial registration:**

The trial was registered before patient enrolment at http://www.chictr.org.cn (ChiCTR2500098527).

## Introduction

Post-acne erythema (PAE) is a common complication following the resolution of inflammatory acne. It is characterized by erythematous macules resulting from the release of inflammatory cytokines such as interleukin-6 (IL-6) and tumor necrosis factor-α (TNF-α) (1). Currently, there is no definitive treatment for PAE (2). Although some lesions may gradually improve over time, persistent facial erythema remains a concern for many patients and can significantly impact their psychological well-being and social functioning (1).

Light and laser-based therapies are among the most frequently used treatments for PAE; however, they generally require multiple sessions and are associated with high costs (3). The substantial expense of laser equipment and consumables also limits the accessibility of these treatments in primary care settings. Several topical agents—such as 0.025% tretinoin, 0.2% brimonidine tartrate, 12% glycolic acid, 5% tranexamic acid solution, and vitamin C formulations—are commonly used for PAE (4, 5). Nonetheless, large-scale, long-term clinical trials have yet to confirm their efficacy and safety (3). Thus, there is an urgent need to identify safe, effective, and practicable treatment options for PAE.

The pathogenesis of PAE has been theorized to involve exogenous stimuli, including topical medications (6). Botulinum toxin, a toxic protein produced by *Clostridium botulinum* includes serotype A (BTX-A), which is a neurotoxic protein that acts on motor nerve endings to inhibit the release, fusion, and docking of acetylcholine. As a result, BTX-A has shown significant therapeutic benefits in conditions such as paralytic strabismus, blepharospasm, delayed muscle palsy, and detrusor hyperreflexia (7–10). BTX-A is also widely employed in cosmetic dermatology for wrinkle reduction, facial contouring, correction of facial deformities, hyperhidrosis, breast reshaping, and suppression of hypertrophic scar formation.

Recent evidence suggests that BTX-A possesses a range of biological properties, including the inhibition of fibroblast proliferation and differentiation, anti-inflammatory effects, antipruritic action, and analgesic activity, indicating its potential as a novel treatment for certain dermatological conditions (7, 11–13). Previous study has demonstrated that BTX-A can significantly improve skin flushing and persistent erythema (14). Bloom et al. (15) administered intradermal BTX-A (15–45 units) to 16 patients with rosacea and observed significant improvement in facial flushing and erythema in 15 patients after 3 months, with no adverse events reported. Similarly, Dayan et al. (16) combined intense pulsed light with subcutaneous BTX-A injections (8–12 units) to treat rosacea, noting marked improvement in facial erythema and flushing within 1 week; the results remained stable at the 3-month follow-up.

Given that PAE is driven by inflammation-induced persistent capillary dilation, and considering the current lack of clinical studies in China evaluating the efficacy and safety of intradermal BTX-A for PAE, this study aimed to investigate the therapeutic potential and safety profile of BTX-A intradermal injection for the treatment of PAE in a Chinese population.

## Materials and methods

### Study design

This was a single-blind, randomized, split-face controlled trial. The outcome assessor responsible for all clinical evaluations and instrumental measurements remained blinded to treatment allocation throughout the study. Due to the inherent nature of the interventions, blinding of the patients and the treating physician was not feasible. The study was approved by the Ethical Committee on Research Involving Human Subjects of the Faculty of Medicine at Henan Provincial People’s Hospital (approval no: 2024–129) and was conducted in accordance with the principles of the 1975 Declaration of Helsinki. This study was registered with the Chinese Clinical Trial Registry (ChiCTR; registration no.: ChiCTR2500098527; available at https://www.chictr.org.cn/searchproj.html). Written informed consent was obtained from all participants.

### Sample size calculation

The sample size for this pilot split-face controlled trial was initially determined based on practical considerations and patient availability during the study period. A *post hoc* power analysis was subsequently performed using G*Power software (version 3.1.9.7) based on the observed effect size for the primary outcome, the Erythema Index (EI), at the 1-month follow-up. Using a paired *t*-test model, a sample size of 30, an effect size (*d*) of 0.75 (derived from the mean difference in EI reduction between groups and the pooled standard deviation), and an alpha error of 0.05, the achieved statistical power was 88%. This confirms that the sample size provided adequate power to detect a clinically significant difference in the primary outcome between the two interventions.

### Patients

Thirty patients with PAE who presented to the Dermatology Outpatient Department of Henan Provincial People’s Hospital between January 2023 and July 2023 were enrolled. Inclusion criteria were as follows: (1) age between 18 and 40 years; (2) presence of PAE on both sides of the face; (3) uniform bilateral facial skin lesions; and (4) stable acne for more than 1 year. Exclusion criteria included: (1) history of keloid or hypertrophic scarring; (2) pregnancy or lactation; (3) recent or planned significant sun exposure; (4) use of photosensitizing drugs or history of photosensitivity; (5) serious systemic diseases, (6) clotting disorders or use of anticoagulants; (7) skin tumors; or (8) immunodeficiency diseases. All enrolled patients confirmed they had not used oral isotretinoin within the 12 months preceding the study, in strict adherence to the exclusion criteria. A history of oral isotretinoin therapy more than 12 months prior to enrollment was reported by 7 patients.

### Randomization and treatment

Randomization and Allocation Concealment: The randomization sequence was generated by an independent statistician using a computer-based random number generator with a 1:1 allocation. The sequence was stratified by the patient’s gender to ensure balance between the potential sides receiving BTX-A. The allocation information was concealed in sequentially numbered, opaque, sealed envelopes. These envelopes were stored securely and were only opened by the treating physician immediately prior to the first procedure for each enrolled patient, thus ensuring allocation concealment. Both groups were treated by the same dermatologist in the same treatment room, and the investigators were blinded to the grouping.

In the experimental group, surface anesthesia was administered using 2% lidocaine ointment for approximately 1 h. A vial of 100 U BTX-A (BOTOX^®^; Allergan Inc., Irvine, CA, United States) was dissolved in 5 mL of normal saline, resulting in a final concentration of 2 U/0.1 mL. This reconstituted 100-unit vial was used as a stock solution for multiple patients; the entire vial was not administered to a single patient. Following skin disinfection with iodophor, the solution was administered using a 1 mL syringe with a 30G, 4 mm needle. The treatment followed a strict intralesional approach: each individual erythematous macule was identified and injected intradermally at a depth of 0.5–1 mm. A volume of 0.05–0.1 mL (containing 1–2 U of BTX-A) was injected into the center of each lesion. Consequently, the number of injection points per cheek was determined by the number of discrete PAE lesions, with a mean of 18 ± 4 points per cheek. The injections were not administered at a fixed distance from each other but were precisely placed within the boundaries of each target macule. This resulted in a typical total dose of 18–36 U per cheek and a total of 36–72 U per patient for the bilateral treatment. The control cheek received three sessions of BBL therapy using the Sciton Joule^®^ platform (Sciton Inc., Palo Alto, CA, United States), with each session spaced 1 month apart. All treatments utilized a 590 nm filter with a fixed spot size of 4.5 × 1.5 cm^2^. The treatment was typically delivered in a single pass. The energy density (fluence) was individualized for each patient based on test spots and the clinical endpoint of mild erythema, ranging from 9 to 11 J/cm^2^. The pulse duration was adjusted accordingly within a range of 10–30 ms. Integrated contact cooling was maintained throughout the procedure to keep the skin surface temperature between 10 and 20 °C. The specific parameters (fluence and pulse duration) used for each patient were documented. All patients were instructed to adhere to the following post-treatment care guidelines: (1) apply sunscreen and moisturizer; (2) avoid contact with water on the treated area for 24 h, (3) avoid oral aminoglycoside antibiotics; and (4) avoid high-temperature facial treatments for 1 week.

### Outcomes

The primary outcome measure was the change from baseline in the Erythema Index (EI) at the 1-month follow-up, as measured by the MPA10 system. Secondary outcomes included the Clinician Erythema Assessment (CEA) score, L*a*b* values (specifically the a* value, which represents the red-green axis), transepidermal water loss (TEWL), sebum secretion, hydration, and adverse events. A researcher not involved in the treatment procedures evaluated patients at baseline (month 0) and at 1, 2, and 3 months after treatment. The CEA was scored on a 5-point scale: (1) 0 (clear skin, no erythema), 1 (almost clear, slight redness), 2 (mild erythema, defined redness), 3 (moderate erythema, marked redness), and 4 (severe erythema, fiery redness). Skin physiological indices (EI, TEWL, sebum secretion, and hydration) were measured using the MPA10 system (Courage and Khazaka, Cologne, Germany), with triplicate measurements taken on both cheeks. The VISIA-CR system (Canfield Scientific, Inc., Parsippany, NJ, United States) and a Colorimeter (Chroma Meter CR-400; Konica Minolta, Inc., Tokyo, Japan) were used as complementary tools to provide a comprehensive assessment of erythema. The VISIA offers standardized photographic documentation and an automated ‘Red Area’ analysis, while the Colorimeter provides objective, quantitative colorimetric data. To ensure measurement consistency, all measurements with both the MPA10 and the Colorimeter were taken in a temperature- and humidity-controlled room after patients had acclimated for 20 min. The same calibrated instruments were used throughout the study, and measurements were performed on the same pre-marked facial areas at each visit by the same trained investigator. Pain associated with the injection therapy was evaluated using a visual analog scale (VAS) ranging from 0 (no pain) to 10 (worst imaginable pain). Adverse reactions, including allergic responses, facial paralysis, erythema, edema, and pigmentation, were also recorded.

### Statistical analysis

The normality of continuous data was assessed using the Shapiro–Wilk test. For comparisons of repeated measures between the two treatment groups, a linear mixed-model analysis was employed. The model included fixed effects for treatment, time, and the treatment-by-time interaction, with subject included as a random effect to account for within-subject correlations. This model is robust to missing data. A significant interaction term indicated that the treatment effect differed over time, prompting simple effect analyses (pairwise comparisons between groups at each timepoint, and within each group over time). For all key outcomes from baseline to follow-up timepoints, the mean difference along with the 95% confidence interval (CI) are reported. The Bonferroni method was applied to adjust *p*-values for these multiple pairwise comparisons. All tests were two-tailed, with a significance level of 0.05. Statistical analyses were performed using SPSS (version 20.0; IBM Corp., Armonk, NY, United States).

## Results

### Baseline characteristics

**Figure 1:**
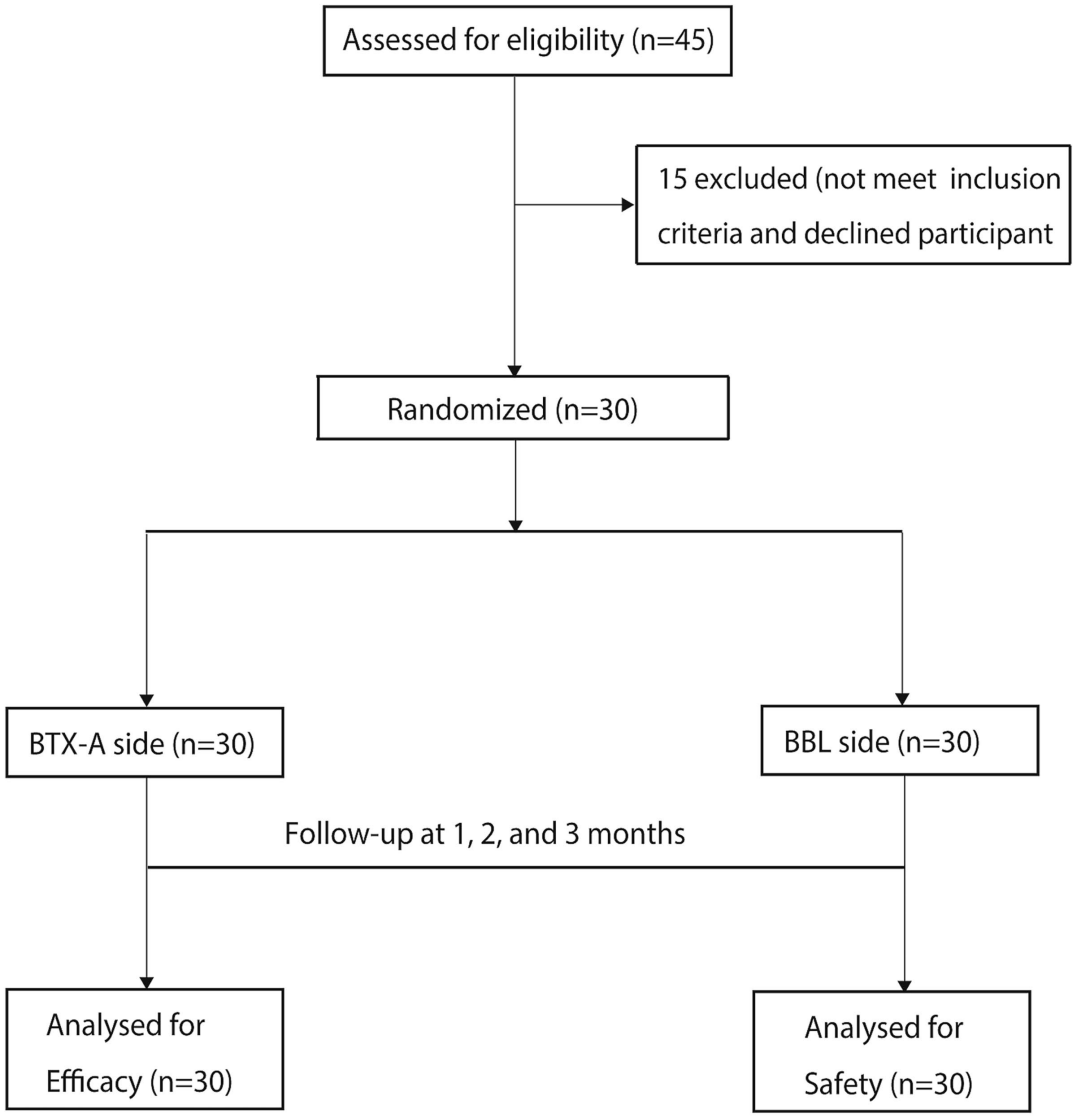
CONSORT flow diagram of participant recruitment and follow-up. The diagram illustrates the enrollment, allocation, follow-up, and analysis of participants in the split-face randomized controlled trial. It details the number of patients assessed for eligibility, excluded (with reasons), randomized, allocated to each intervention (BTX-A on one cheek, BBL on the contralateral cheek), completed follow-up, and included in the analysis.

The flow of participants through each stage of the trial is summarized in Figure 1. Briefly, a total of 45 patients were assessed for eligibility between January and July 2023. Of these, 15 were excluded. The remaining 30 patients were enrolled and randomized. All 30 patients received the allocated intervention and were included in the final analysis at the 3-month endpoint. The study population comprised 30 patients (females: 53.3%; mean age: 24.5 ± 4.8 years). Baseline characteristics, including skin physiological indices, CEA scores, and L*a*b* values, showed no significant differences between the experimental and control groups (all *p* > 0.05, Table 1).

**Table 1:** Baseline demographic and clinical characteristics of the study participants.

Characteristics	BTX-A group (*N* = 30)	BBL group (*N* = 30)	*p*-value
Age (years)			
Mean ± SD	24.5 ± 4.8	/	/
Range	18–35	/	/
Gender, *n* (%)			
Male	14 (46.7)	/	/
Female	16 (53.3)	/	/
Skin physiological indexes, mean ± SD			
Melanin content	156.6 ± 38.2	161.9 ± 40.4	0.604
Erythema index	384.6 ± 71.0	404.5 ± 77.7	0.303
TEWL	25.5 ± 10.4	28.0 ± 11.4	0.396
Sebum secretion	43.6 ± 18.8	37.0 ± 13.7	0.123
Skin hydration	44.8 ± 11.7	45.2 ± 10.4	0.882
CEA score, mean ± SD	2.73 ± 0.74	2.77 ± 0.77	0.865
L*a*b* values, mean ± SD			
L*	59.1 ± 3.8	59.9 ± 3.4	0.444
a*	18.2 ± 4.8	17.9 ± 5.4	0.876

Values are presented as mean ± standard deviation, median (interquartile range), or number (percentage), as appropriate. SD, standard deviation; TEWL, transepidermal water loss; CEA, clinician erythema assessment; BTX-A, Botulinum Toxin Type A; BBL, Broadband Light.

### Skin-related indices at 1, 2, and 3 months

At the 1-month follow-up, the experimental group showed a significantly greater reduction in the EI compared to the control group (−67.2 vs. −37.0; *p* = 0.001). The decrease in sebum secretion was also significantly greater in the experimental group at 1 month (−15.0 vs. −4.0; *p* < 0.001), 2 months (−17.5 vs. −12.0; *p* = 0.020), and 3 months (−21.0 vs. −13.2; *p* = 0.002). Similarly, the mean CEA score decreased significantly more in the experimental group than in the control group at 1 month (−0.80 vs. −0.50; *p* = 0.032) and 2 months (−1.40 vs. −1.03; *p* = 0.014). Regarding colorimetric evaluation, the a* value decreased significantly more in the experimental group at 1 month (−3.79 vs. −1.23; *p* < 0.001) and 3 months (−4.78 vs. −3.29; *p* = 0.019) (Table 2).

**Table 2:** Changes in primary and secondary outcomes from baseline to follow-up timepoints in the BTX-A and BBL groups.

Characteristics	BTX-A group (*N* = 30)	BBL group (*N* = 30)	*P*-value
Melanin content			
Month 1	−13.8 (−22.8, −8.8)	−12.4 (−21.3, −8.2)	0.663
Month 2	−16.7 (−33.0, −7.4)	−24.1 (−41.5, −15.8)	0.117
Month 3	−22.5 (−40.2, −10.0)	−31.9 (−51.6, −22.9)	0.056
Erythema index			
Month 1	−67.2 (−109.6, −56.5)	−37.0 (−67.3, −20.5)	**0.001**
Month 2	−76.2 (−102.4, −47.2)	−60.2 (−99.6, −44.4)	0.647
Month 3	−65.2 (−134.0, −22.3)	−70.5 (−117.1, −45.6)	0.595
TEWL			
Month 1	−4.6 (−9.7, −2.7)	−6.2 (−8.5, −3.2)	0.773
Month 2	−7.7 (−18.4, −1.6)	−8.9 (−16.8, −5.5)	0.344
Month 3	−11.6 (−19.7, −5.6)	−11.4 (−20.2, −5.3)	0.877
Sebum secretion			
Month 1	−15.0 (−25.0, −11.5)	−4.0 (−6.0, −3.0)	**<0.001**
Month 2	−17.5 (−28.5, −11.0)	−12.0 (−16.2, −6.2)	**0.020**
Month 3	−21.0 (−30.3, −13.5)	−13.2 (−17.3, −6.3)	**0.002**
Skin hydration			
Month 1	6.9 (3.0, 10.4)	5.6 (1.7, 11.0)	0.796
Month 2	9.9 (4.1, 16.3)	10.8 (3.5, 18.0)	0.882
Month 3	10.0 (4.9, 21.8)	10.5 (4.8, 22.3)	0.756
CEA score			
Month 1	−0.80 ± 0.48	−0.50 ± 0.57	**0.032**
Month 2	−1.40 ± 0.56	−1.03 ± 0.56	**0.014**
Month 3	−1.63 ± 0.72	−1.47 ± 0.63	0.343
L*a*b* values			
a* value			
Month 1	−3.79 (−4.81, −3.02)	−1.23 (−1.74, −0.90)	**<0.001**
Month 2	−3.63 (−5.45, −2.85)	−3.52 (−4.62, −2.09)	0.429
Month 3	−4.78 (−6.77, −3.23)	−3.29 (−4.68, −2.61)	**0.019**

Data are presented as mean ± standard deviation. *P*-values are derived from linear mixed-model analysis with Bonferroni correction for *post hoc* pairwise comparisons. BTX-A, Botulinum Toxin Type A; BBL, Broadband Light. Bold values indicated *P*<0.05.

### Skin physiological index

**Figure 2:**
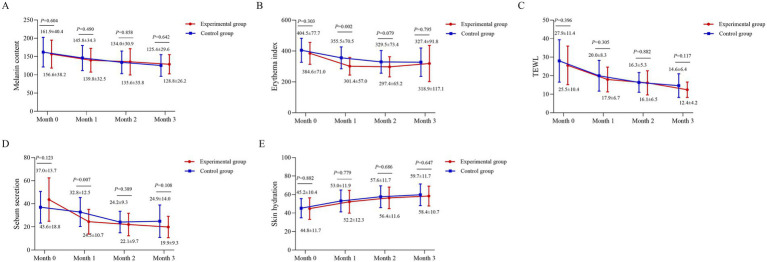
Longitudinal changes in skin physiological indices. **(A)** Melanin content, **(B)** Erythema Index (EI, arbitrary units), **(C)** transepidermal water loss (TEWL, g/m^2^/h), **(D)** sebum secretion (μg/cm^2^), and **(E)** hydration (arbitrary units) are shown for the BTX-A and BBL-treated sides across the study period. Measurements were obtained using the MPA10 system (Courage and Khazaka, Cologne, Germany). Data points represent mean values, and error bars represent standard deviation. A linear mixed-model was used for analysis, with subject as a random effect and fixed effects for treatment, time, and their interaction. Between-group comparisons at each time point were conducted based on the model’s estimates.

Compared to baseline, both groups exhibited a decreasing trend in melanin content, EI, TEWL, and sebum secretion, alongside an increasing trend in skin hydration (Figures 2A–E). No significant intergroup differences were observed in melanin content, TEWL, or skin hydration at 0, 1, 2, and 3 months (*p* > 0.05) (Figures 2A,C,E). The EI was significantly lower in the experimental group than in the control group at month 1 (301.4 vs. 355.5; *p* = 0.002), though no significant differences were noted at other time points (*p* > 0.05) (Figure 2B). Sebum secretion was also significantly lower in the experimental group at month 1 (24.5 vs. 32.8; *p* = 0.007), with no significant differences at other assessments (*p* > 0.05) (Figure 2D).

### CEA score and L*a*b* value (a*)

**Figure 3:**
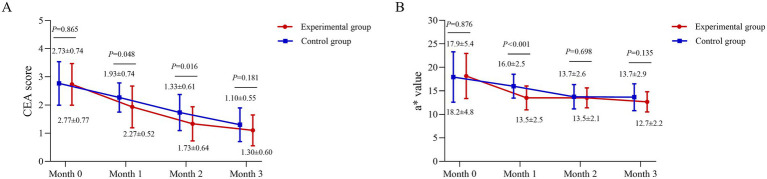
Temporal profile of clinical erythema and colorimetric improvement. **(A)** Clinician Erythema Assessment (CEA) scores (scale 0–4) and **(B)** Colorimeter a* values (representing the red-green axis) for the BTX-A and BBL-treated sides across the study period. The a* value was measured using a Colorimeter (Chroma Meter CR-400; Konica Minolta, Inc., Tokyo, Japan). Data are presented as mean ± standard deviation. A linear mixed-model was used for analysis. Between-group comparisons at each time point were derived from the model.

At month 1, the CEA score was 2.27 ± 0.52 in the experimental group and 1.93 ± 0.74 in the control group (*p* = 0.048). At month 2, the scores were 1.73 ± 0.64 and 1.33 ± 0.61, respectively (*p* = 0.016). No significant differences were observed at baseline or month 3 (*p* > 0.05) (Figure 3A).

The a* value were assessed at months 0, 1, 2, and 3. Baseline values were comparable between groups (*p* > 0.05). At month 1, the experimental group showed a significantly lower a* value (13.5 ± 2.5) than the control group (16.0 ± 2.5; *p* < 0.001). This difference was not maintained at later time points, with no significant disparities at months 2 or 3 (both *p* > 0.05) (Figure 3B).

### VAS score and satisfaction score

**Figure 4:**
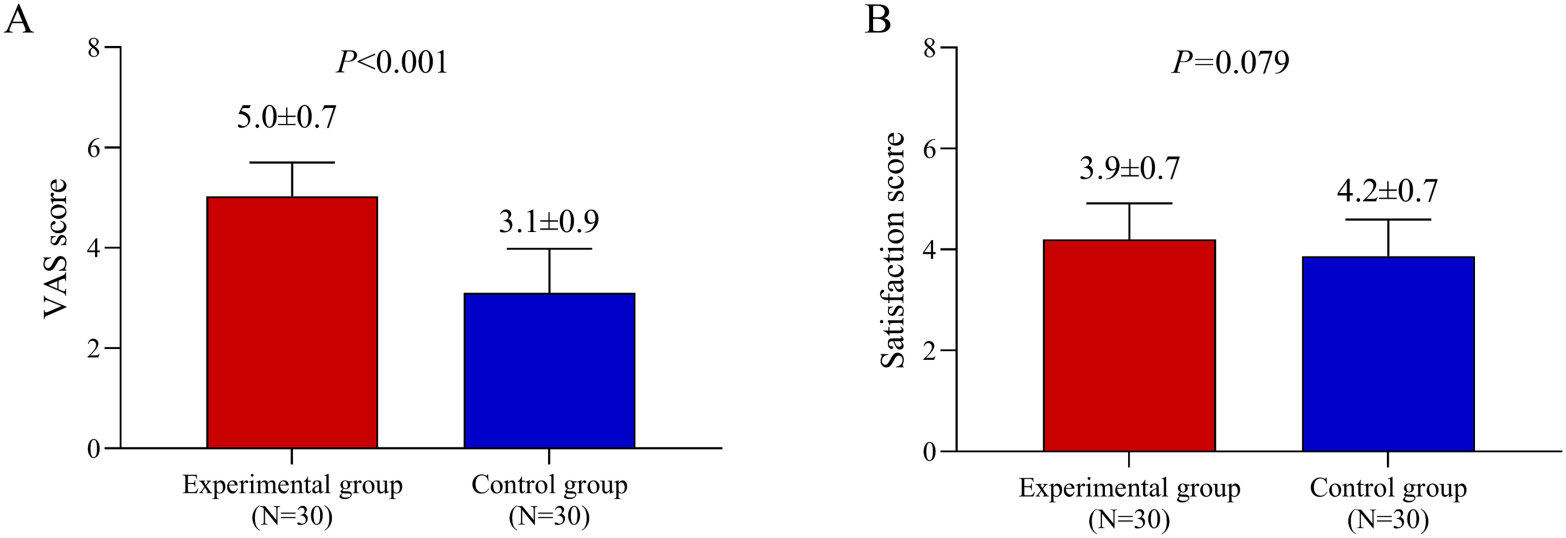
Patient-reported outcomes of pain and satisfaction. **(A)** Visual Analog Scale (VAS) pain scores (range 0–10, where 0 is no pain and 10 is the worst pain imaginable) reported immediately after the first treatment session. **(B)** Patient satisfaction scores at the 3-month follow-up. Data are presented as mean ± standard deviation. Comparisons between the BTX-A and BBL sides were performed using a paired *t*-test.

The VAS score for pain or discomfort was significantly higher in the experimental group than in the control group (5.0 vs. 3.1; *p* < 0.001). In contrast, satisfaction scores did not differ significantly between the groups (*p* = 0.079) (Figure 4).

### Case presentation

**Figure 5:**
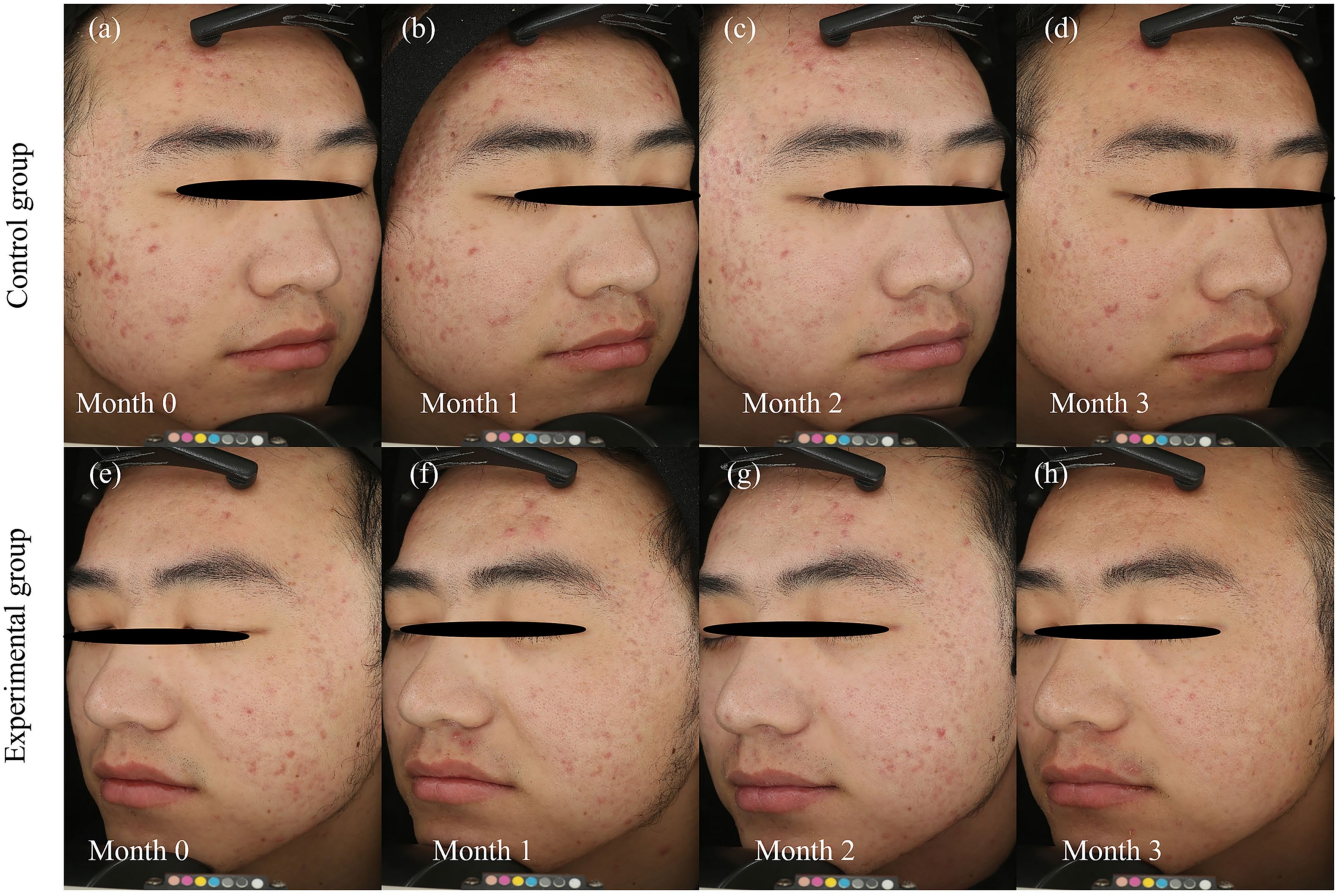
Dynamic resolution of post-acne erythema following BTX-A and BBL treatments. Serial clinical photographs document the temporal evolution of post-acne erythema (PAE) over the 3-month study period in a representative patient. BBL Group: **(a)** baseline (month 0), before the first BBL treatment, **(b)** follow-up at Month 1, **(c)** follow-up at month 2, **(d)** follow-up at month 3. BTX-A group: **(e)** baseline (month 0), before BTX-A injection, **(f)** follow-up at month 1, **(g)** follow-up at month 2, **(h)** follow-up at month 3. Photographs were captured using the VISIA-CR system (Canfield Scientific, Inc., Parsippany, NJ, United States). The photographic series demonstrates a progressive reduction in the intensity and area of erythema on both sides. The BTX-A-treated side **(e–h)** exhibits a more rapid and pronounced improvement, particularly evident in the early follow-up periods, and sustained throughout the 3-month duration. In contrast, the BBL-treated side **(a–d)** shows a more gradual improvement, consistent with the cumulative effect expected from a multi-session light-based therapy.

**Figure 6:**
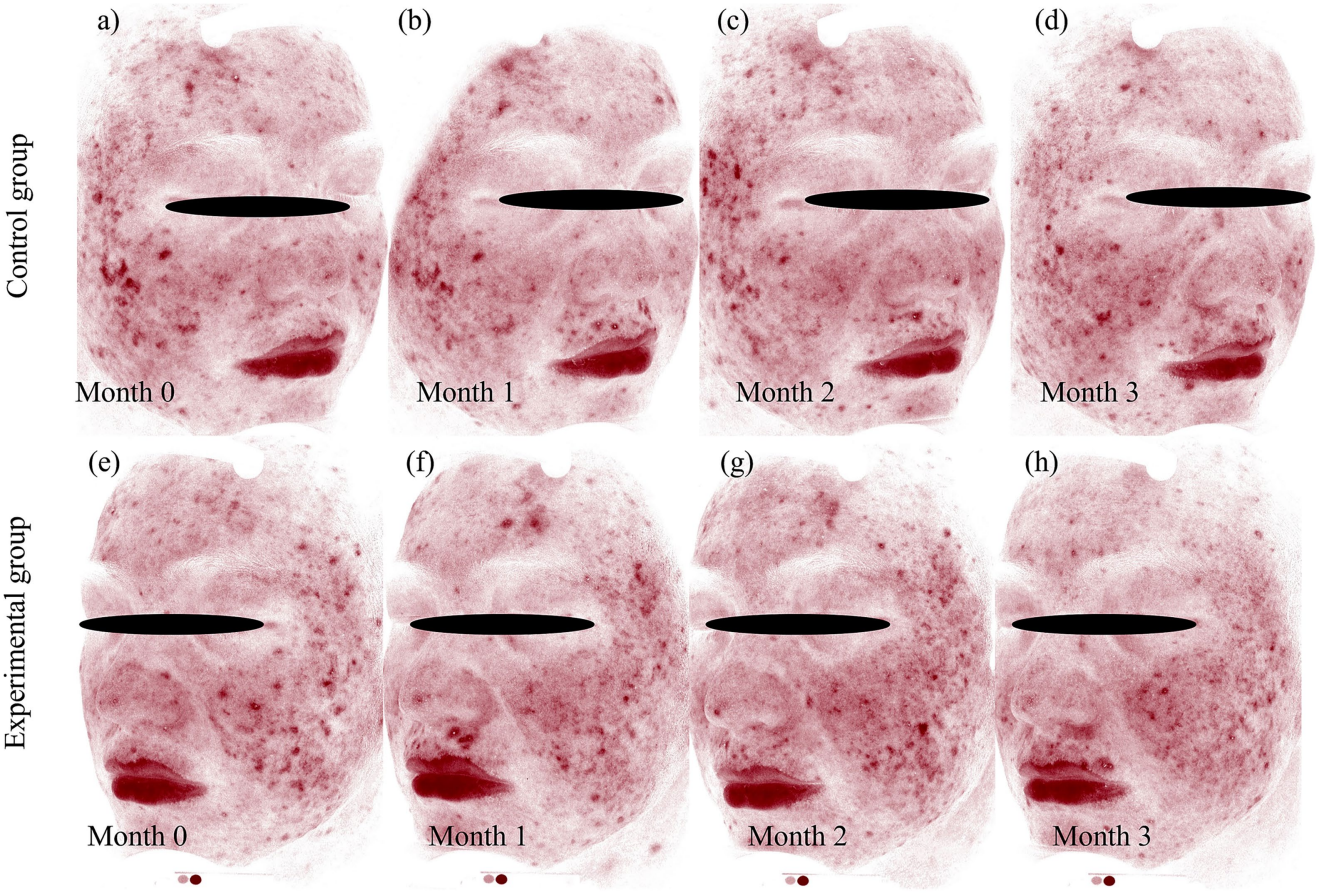
VISIA-CR quantitative analysis of erythema resolution following BTX-A and BBL treatments. Standardized VISIA-CR system images with the “Red Area” feature highlighted, providing objective quantification of erythema changes corresponding to the clinical photographs. BBL Group: **(a)** baseline (month 0), showing widespread red areas, **(b)** month 1, demonstrating initial reduction in erythema, **(c)** month 2, showing continued clearance, **(d)**: month 3, with significant but incomplete resolution. BTX-A group: **(e)** baseline (month 0), comparable to the control side, **(f)** month 1, showing a marked and rapid decrease in red areas, **(g)** month 2, with near-complete resolution, **(h)** month 3, sustaining excellent clearance. Photographs were captured using the VISIA-CR system (Canfield Scientific, Inc., Parsippany, NJ, United States).

Representative cases from the experimental and control groups are shown in Figures 5, 6, illustrating improvements in erythema before and after treatment. Longitudinal photographs of both facial sides were obtained at 0, 1, 2, and 3 months. The right cheek received BBL treatment, and the left cheek received BTX-A. Both sides showed notable improvement in the extent and severity of erythema, with more substantial changes observed on the BTX-A–treated side.

## Discussion

Acne is the most common skin condition, affecting approximately 80% of teenagers and young adults worldwide. PAE is a common sequela of acne-induced inflammation, particularly in individuals with fair skin (2). Currently, there are no specific drugs for PAE and some acne medications can exacerbate it (17). Previous studies confirmed that BTX-A significantly improves skin flushing and persistent erythema by inhibiting the release, fusion, and docking of acetylcholine (14). However, no clinical studies in China have confirmed the efficacy and safety of intradermal BTX-A injection therapy for PAE. To the best of our knowledge, this is the first study to evaluate the efficacy and safety of intradermal BTX-A injection therapy for PAE in China. We found that BTX-A treatment was more effective than BBL in reducing skin-related indicators such as CEA and a* values. However, the pain level in the experimental group was significantly higher than that in the control group, and there was no significant difference in satisfaction scores between the two groups.

The pronounced reduction in erythema observed following intradermal BTX-A injections can be attributed to its direct and indirect effects on the cutaneous vasculature. Beyond its well-known action of inhibiting acetylcholine release at the neuromuscular junction, BTX-A exerts potent effects on the autonomic cholinergic innervation of cutaneous blood vessels. By blocking acetylcholine, BTX-A inhibits the endothelial nitric oxide synthase-dependent pathway, leading to a reduction in nitric oxide production, a key mediator of vasodilation (15–18). Furthermore, growing evidence indicates that BTX-A can suppress the release of other vasoactive neuropeptides from sensory and autonomic nerves, such as calcitonin gene-related peptide (CGRP), substance P, and vasoactive intestinal peptide (19–22). This multi-faceted inhibition of vasodilatory signals results in sustained vascular contraction, reducing blood flow and thereby diminishing the appearance of persistent erythema. The anti-inflammatory properties of BTX-A, potentially mediated through the downregulation of pro-inflammatory cytokines, may also contribute to resolving the underlying inflammatory component of PAE (6, 23–26).

BTX-A significantly improves skin-related indicators and physiological indices in patients with PAE. This may be due to its inhibitory effect on vasodilating neuropeptides such as acetylcholine and vasoactive intestinal peptides, and its interference with the release of neurotransmitters such as substance P and CGRP. Additionally, BTX-A can suppress vascular endothelial growth factor expression by modulating inflammation (27, 28). In addition, the study found that TEWL and sebum secretion decreased, whereas skin hydration increased in the experimental group. These results were consistent with those of other studies. Studies have shown that intradermal injection of BTX-A reduces sebum secretion and increases skin hydration over a 12-week period (18, 29). The mechanism underlying reduced sebum secretion is thought to involve BTX-A blocking the release of acetylcholine at the neuromuscular junction and its receptor binding in postsynaptic cells (30). In terms of patient satisfaction, the treatment achieved high levels without significant side effects (29). The significantly higher VAS pain scores associated with the intradermal BTX-A injections, contrasted with the non-significant difference in final satisfaction scores, presents a compelling clinical finding. This suggests that from the patient’s perspective, the trade-off between a brief, more painful procedure and the subsequent, pronounced, and sustained improvement in erythema is acceptable. The high satisfaction likely reflects the value patients place on the efficacy and durability of the outcome, which may outweigh the memory of transient procedural discomfort. From a clinical implementation standpoint, this trade-off is highly relevant. Patients seeking a rapid and effective solution for PAE may reasonably accept a single, more painful session if it yields superior and longer-lasting results compared to multiple, less uncomfortable sessions of light-based therapy. To further improve the patient experience and minimize the procedural barrier, several simple and effective mitigation strategies can be employed. These include optimizing analgesia by prolonging the application time of topical anesthetics, using finer-gauge needles for the injections, and potentially employing vibration or cold devices for distraction analgesia. The application of a nerve block, while more involved, represents another option for maximizing patient comfort. Future clinical protocols should formally incorporate and evaluate such strategies to enhance tolerability without compromising efficacy.

The observed improvements in our primary and secondary outcomes were not only statistically significant but also likely to be clinically relevant. For instance, the reduction in the CEA score in the BTX-A group exceeded 1 point at the 2-month follow-up. A change of this magnitude on a 5-point clinician-rated scale is often considered a substantial and perceptible improvement in clinical practice. Similarly, the reduction in the a* value by approximately 4 units in the BTX-A group represents a **pronounced decrease in redness, as a change of 1.5–2.0 a* units has been suggested in previous colorimetric studies to be visually detectable **. Therefore, the improvements we documented are considerable and likely to be perceptible to both patients and clinicians, underscoring the potential of intradermal BTX-A as an impactful treatment modality for PAE.

A key aspect of our study design is the comparison of a single session of intradermal BTX-A against three sessions of BBL. We acknowledge this asymmetry in the number of treatments. This design was chosen for several reasons. First, it reflects the distinct mechanisms and temporal profiles of the two modalities. BTX-A is known to have a prolonged biological effect, with studies showing benefits for facial erythema lasting over 3 months from a single treatment session (15, 16). In contrast, BBL and other light-based therapies typically require multiple, cumulative sessions to achieve optimal results (3). Our aim was to compare a typical clinical course of BBL against a typical single administration of BTX-A. The sustained improvement observed in the BTX-A group over the entire 3-month study period, often surpassing the multi-session BBL regimen, underscores the potent and durable effect of the neurotoxin for PAE. Nonetheless, this design does not allow for a direct comparison of the maximal potential efficacy of each modality when both are administered in their multi-session optimal regimens. Future studies comparing multiple BTX-A sessions to multiple BBL sessions would be valuable to further explore this question.

This study has several limitations. First, the relatively small sample size (*n* = 30) from a single tertiary hospital limits the generalizability of our findings. Although our *post hoc* analysis indicated sufficient power for the primary outcome, this sample size may have limited our ability to detect smaller but still clinically meaningful effects for some secondary outcomes and to perform more comprehensive subgroup analyses. The strict inclusion and exclusion criteria, while necessary for internal validity, further contributed to a homogeneous sample, potentially limiting the applicability of our findings to all patients with PAE. Future multicenter studies with larger, more diverse sample sizes are needed to validate these findings and explore prognostic factors. Second, while the VISIA-CR system was used for standardized photographic documentation and its ‘Red Area’ analysis for qualitative illustration, a comprehensive quantitative analysis of the VISIA ‘Red Area’ metric for all patients was not a pre-specified endpoint in our statistical analysis plan. Therefore, the VISIA data presented in this study should be interpreted as supportive, qualitative documentation of clinical improvement. The primary quantitative analyses for erythema were validly based on the pre-specified and highly objective MPA10 Erythema Index and colorimeter a* value. Future studies would benefit from prospectively incorporating the quantitative analysis of standardized photographs, such as the VISIA ‘Red Area’ or other photographic analysis tools, as a core outcome measure. Third, the 3-month follow-up period primarily demonstrates short-term efficacy. The durability of the therapeutic effect and the potential for recurrence beyond 3 months remain unknown. Furthermore, the optimal injection interval and the long-term safety profile of repeated intradermal BTX-A injections for PAE warrant investigation in future studies with extended follow-up durations. Fourth, as a single-center study conducted at a tertiary hospital, the patient population may not be fully representative of the broader community, which affects the generalizability of our results. The strict inclusion and exclusion criteria, while necessary for internal validity, further contributed to a homogeneous sample, potentially limiting the applicability of our findings to all patients with PAE. Future multicenter studies with larger sample sizes are needed to validate these findings and explore prognostic factors.

## Conclusion

In this split-face controlled trial, intradermal BTX-A demonstrated superior efficacy to BBL in improving both clinical and instrumental measures of PAE, with a favorable tolerability profile. The sustained therapeutic effect over the 3-month study period supports the potential of BTX-A as an effective and practical short-term treatment option for PAE. Notably, a single session of BTX-A achieved more pronounced and rapid improvement than multiple BBL sessions. However, it is important to note that our findings demonstrate efficacy within a three-month window, and the long-term durability is not established by this study. Given the limitations of sample size and single-center design, future research should prioritize larger-scale, multicenter trials with extended follow-up to validate these findings, assess long-term safety and efficacy, and facilitate the generalizability of the results. Further investigation is also warranted to optimize treatment protocols, including determination of ideal dosing, injection intervals, and potential combination therapies, to facilitate personalized management strategies in clinical practice.

## Data Availability

The raw data supporting the conclusions of this article will be made available by the authors, without undue reservation.
